# Indents

**Published:** 1922-07

**Authors:** 


### INDENTS

**☞Brief Articles of Technical or Scientific Value will receive notice and comment. Contributions invited.**

---

TO POLISH RUBBER DENTURES. After the plate is filed, scraped and sandpapered, instead of the felt cone use a cone made of a wine cork, one end of which is screwed into a chuck and the other end rounded with a file while the latter is revolving. On this use pumice in which has been incorporated some emery dust moistened. This cuts much faster than pumice alone, and will take out the scratches left by the sandpaper very rapidly. Follow this with pumice without the emery, then use whiting moistened with ammonia.

If a fine polish is required, employ prepared chalk and ammonia after the whiting. The commercial whiting contains considerable grit, and while it will polish it leaves many shallow scratches which the fine chalk eliminates.—F. J. Patterson, *Dental Facts.*

---

THE CARE OF THE TEETH. While the article is elementary in content, it contains matter of general interest. The splendid teeth and ''reinforced'' alveoli of the Esquimaux appear to be due entirely to the use he makes of them not only for eating but as a handy tool for routine work. While the baby at birth is commonly spoken of as toothless, his gums contain not only preexistent milk teeth, but the germs of the permanent teeth. The six year molar is the most important tooth in the head and the most neglected. One may learn much about the proper use of the toothbrush by smearing the nails with clay or vaseline, and then removing this artificial coating with the brush. The keynote of cleansing the teeth, aside from keeping away germ life, is that every accessible surface should be polished.

Under pyorrhea it is stated that accumulation of tartar where teeth and gums join is a contributing cause but can not produce the condition alone. Other substances can produce irritation and infection of the gums. There is no specific and the only treatment is to remove all of the foreign matter and then polish the exposed portions of the teeth. All depends on systematic cooperation of dentist and patient with quarterly consultations.

Under focal infection the X-ray plate must be the criterion and it will reveal the occurrence of pus collections and pockets filled with putrid detritus which are a menace to health. It does not follow that these are the sole cause of rheumatism or even perhaps the only factor in a given case, but they are certainly a common factor both in certain diseases and in the ill health of the individual.—*Public Health Reports,* November, 1921.

---

CANDY. It would be a great relief if, in the United States of America, some reformer at some time would be able to add the word "do" to his vocabulary and eliminate the word "don't." Whenever some "solemncholy" individual feels dissatisfied with the world, he immediately gets up an organization to have another "don't" slipped into the legal machinery of this long suffering country.

Away back in the dawn of history, somebody made the alleged discovery that decay of the teeth was due to candy. It is quite probable that candy and vegetables and meats and fruits and grain and water, to say nothing of eggs, fish and other food products, contribute to the decay of the teeth. In fact, if all foods were eliminated for a sufficient length of time there would be no further decay of anybody's teeth; but why hit on candy?

Any sane person realizes that overeating will secondarily have a deleterious effect upon the teeth and mouth. It does not make any difference what particular type of food is indulged in to the point of overloading the stomach. Moderation in diet is the thing to be aimed at.

If it were possible for candy to be cut entirely out of the menu, the much desired absence of decay would not be gained thereby. This was amply shown in the mouths of children in France and England during the war. Many thousands of children not only had no candy but had no sugar in their diet and their teeth decayed just the same. The way to prevent caries is by a rational, well-balanced diet and by the proper and thorough brushing of the teeth followed by first-class dental attention when necessary.

The children of the future will be so surrounded by ''verboten'' that their natural inclinations will be lost in the maze of the artificial protection that we are building up for them.

Every group of reformers desires to rush into public print to tell the people what not to do. One of these days we will probably hear the newsboys crying in the streets, ''Extra! Extra! The latest list of forbidden joys!''

The prime idea in this type of legislation is that it may eventually lead to the total abolition of rent, because, when we all are finally convicted of breaking the reform laws, the government can build apartment houses with iron doors and we can serve our terms at home.—*Oral Hygiene.*

---

Observations on Dental Radiography. A decade or two ago it was estimated that less than 5 per cent. of the people of the United States were educated to the needs of dental service, but in the past few years both the lay and professional magazines have given recognition to the importance of Dental Health. Dental inspection in our public schools, the work of Rosenow and others calling attention to the effects of oral foci of infection from diseased teeth, and higher standards of dental education have all contributed to the popularity of dentistry, and it is now estimated that 20 per cent. of our people are seeking services of dentists.

All progressive members of the profession of dentistry and medicine realize the importance of proper and thorough examination of the mouth and teeth as an essential part in general physical diagnosis. Proper and thorough examination of the mouth and teeth can not be made without the use of the X-ray.

The use of the X-ray is of little value unless the resulting radiograms are made by some one who understands dental anatomy thoroughly and has acquired that high degree of skill or technique necessary to secure a radiogram, possessing diagnostic value.

A truly diagnostic radiogram is of little value unless properly read.

Let us analyze these statements briefly:

"Proper and thorough examination of the mouth and teeth can not be made without the use of the X-ray." That the use of the X-ray in dentistry is indispensable is recognized by every member of the profession to a certain degree. The full measure of its usefulness is not fully realized by some, however.

There are some practitioners, who insist upon having radiograms of the entire mouth before beginning work on a new patient. The satisfactory results they have obtained in planning their work, in avoiding future trouble and in holding the confidence of their patients has amply justified their methods.

Others have all devital teeth radiographed and obtain nearly as good results. They, however, sometimes miss some of the obscure conditions that may mean the success or failure of their efforts.

The following is an example of a case in practice:

Case 916—Male, 35 years of age. Neuralgia, reflex neuroses, etc. Sent to have devital upper teeth radiographed. Anterior teeth from right to left bicuspids had been removed and replaced with a bridge. Was instructed to radiograph upper right molars and bicuspids and upper left molars and bicuspids. Suggested necessity of radiographing right and left cuspid region for possible broken root or impacted teeth, but not thought necessary by patient or dentist.

Radiograms showed piece of root of molar under bridge, with radiolucent area surrounding same, also part of root of upper right cuspid, probably unerupted. It was then thought necessary to radiograph upper right and left cuspid, lateral and central regions. Resulting radiograms disclosed unerupted, impacted right and left cuspids with considerable destruction of process around crowns of teeth and fistula.—*Northwestern Journal.*

---

THE VALUE OF CORRECT RADIOGRAPHIC INTERPRETATION.

It has frequently and correctly been asserted that the radiograph can not be relied upon as a basis of diagnosis unless coupled with a clinical examination. Yet it is equally true that in many instances the clinical findings would be unreliable without a radiographic check-up. Indeed, this is so true that one may state unequivocally that many clinical facts can only be discovered with the "X-ray eye" which can "see through solids."

Even so simple a matter as the removal of a pulp may be misinterpreted. At the next sitting after supposed complete removal the exploring broach causes intense pain. Has it passed through the apical foramen and is the pain from the periapical tissues? Or was there a residuum of pulp left in, so that it responds to the touch of a broach? Who has fingers so deft that he can diagnosticate such a case by clinical examination alone? But insert a broach carefully until the patient just feels it, then make a radiograph and the truth is revealed. If the broach does not reach the apical foramen we know at once that pulp tissue is still present.

In the earlier days of radiodontia, radiographic examination was largely restricted to such facts; such as the distance to which a diagnostic wire had been passed; whether or not a gutta percha filling reached the apex, etc., etc. A little later the focal infection fad came upon us with full force and we began to focus our attention on the area just about the apex. A dark spot on a film was called a "shadow," which it never is, and such "shadows" were interpreted as abscesses, granulomas or cysts. This was the beginning of our radiographic studies of the altered appearances of bone that might be caused by infections or other pathologic conditions.

We are now entering a period where we shall study the entire alveolar process; where we must acquire a knowledge of the radiographic appearances of normal, healthy bone, and also of variations from the normal that may be caused by factors physiologic or pathologic.

This study of bone, its variation in health and disease, and the consequent alterations of the radiographic picture is intensely interesting and of fundamental importance both to the dental diagnostician and to the dental practitioner.

The medical profession, and to a large extent the dental men, are explaining the etiology of an increasing number of body ills as originating in focal infections about the teeth. Unfortunately the treatment is nearly always the same. The supposed presence of apical infection as disclosed by a radiographic film is sufficient excuse for the removal of the tooth. This emphasizes the importance of correct interpretation of radiographs.

When the radiodontist shall become entirely familiar with the radiographic records made by alveolar bone both normal and abnormal; by bone that is physiologic and bone that is pathologic; when he can differentiate between a "granuloma" that is forming, and one that is being slowly destroyed and replaced by bone; when he recognizes all the changes that may occur both in the pericementum and in the lamina dura, or peridental lamella, to adopt Pollia's term, then may we not hope for the arrival of a day when radiographic interpretation may be dependable even without the clinical examination, however valuable the latter always will be? In pursuance of this possibility we are more than pleased at the present stage of our Discussion by Correspondence, which we think has assumed a great degree of interest and importance.

In the April issue Dr. McCall presented a number of radiographs with which he undertook to show that certain alterations in the peridental lamella which the present writer had suggested might be considered as stigmata of fixed bridgework, likewise are to be found in connection with traumatic occlusion in the absence of bridgework.

*Dr. Pollia's Discussion of Dr. McCall's Radiographs*

In this issue Dr. Joseph Pollia of San Francisco replies to Dr. McCall, and declares that he can differentiate with the radiograph the conditions resulting from traumatic occlusion, from those found in conjunction with fixed bridgework. If this theory can be substantiated and if it can be so clearly defined that any good radiodontist can likewise make the interpretations, it certainly will be a great step in advance. At present, however it does seem to be complicated. Granting that Dr. Pollia may be correct, so far as the fundamental principles are concerned, yet it would appear that traumatic occlusion in conjunction with a suppurative periclasia, might very closely resemble the picture of fixed bridgework cases, where the bridge pontics are likewise in traumatic occlusion.

Nevertheless the communication from Dr. Pollia is along correct lines, and is of special interest since he repeats Dr. McCall's own radiographs, giving us his (Pollia's) interpretations in juxta-position with Dr. Mc-

Call's. This we believe has not been heretofore done, and is most instructive. Our one regret is that the differentiations, so clearly seen in the actual radiographic films, are so much less discernible when we must first make a print from the film, then make a half tone reproduction, and lastly print the same on a printing press, with all the chances of fogging the nice details with too much or too little ink. However we think this study of radiographic interpretation so important to the future progress of dentistry as well as to the welfare of future patients that we are doing all possible to improve our published reproductions of radiographic films.—Dr. R. Ottolengui, in *Dental Items of Interest.*

